# Clinical and Pathological Study of Tumor Border Invasion—Is Narrow Resection Margin Acceptable in Hepatoblastoma Surgery?

**DOI:** 10.3389/fmed.2020.00059

**Published:** 2020-03-04

**Authors:** Gang Shen, Linlin Wu, Jie Zhao, Bin Wei, Xianjun Zhou, Feifei Wang, Jie Liu, Qian Dong

**Affiliations:** ^1^Department of Pediatric Surgery, The Affiliated Hospital of Qingdao University, Qingdao, China; ^2^Department of Pediatric Surgery, Weifang People's Hospital, Weifang, China; ^3^Pathology Group of Shandong Key Laboratory of Digital Medicine and Computer Assisted Surgery, The Affiliated Hospital of Qingdao University, Qingdao, China; ^4^Shandong Key Laboratory of Digital Medicine and Computer Assisted Surgery, The Affiliated Hospital of Qingdao University, Qingdao, China; ^5^Shandong College Collaborative Innovation Center of Digital Medicine in Clinical Treatment and Nutrition Health, Qingdao, China

**Keywords:** hepatoblastoma, border, invasion, pathology, chemotherapy

## Abstract

**Aim:** We aim to study clinically and pathologically whether narrow resection margin (<1 cm) is acceptable in hepatoblastoma surgery.

**Methods:** A total of 42 patients who underwent surgery for hepatoblastoma were selected, and these patients were divided into two groups according to whether or not they underwent preoperative chemotherapy (CHT). The general characteristics of the patients were summarized, the resection margin distance was recorded, and the event-free survival rates were followed up. Pathologically, H&E staining and immunochemical staining were used to study the invasion distance outside the tumor capsule in the tumor border.

**Results:** Clinically, the event-free survival rates were not significantly different between the patients with wide resection margin (>1 cm) and narrow resection margin (<1 cm) of the two groups. Pathologically, the tumor of all 42 patients had capsules surrounding the tumor. Of the patients in Group 1 (without preoperative CHT), 9% (2/22) had micrometastatic cancer nests outside the capsule, and the farthest distance from the cancer nests to the capsule was 4.6 mm. Of the patients in Group 2 (with preoperative CHT), 75% (15/20) showed residual cancer nests in the paratumor liver tissue, and the farthest distance was 9.6 mm; three and two cases, respectively, showed extracapsular intravascular microtumorous thrombi.

**Conclusion:** Clinically and pathologically, narrow resection margin is acceptable in hepatoblastoma surgery.

## Introduction

Hepatoblastoma is the most common primary malignant liver tumor in children. It accounts for 50–60% of primary malignant liver tumors and 25–45% of all liver tumors. With the establishment of the PRETEXT system as well as chemotherapy (CHT) and surgery treatment model (1), and following multidisciplinary comprehensive treatment, the 5-year survival rate of children with hepatoblastoma can reach more than 60% (2, 3).

Complete surgical resection is the most important step in the comprehensive treatment process and provides the only practical possibility for long-term survival (4). However, the range of resection remains controversial. Most scholars believe that for patients with PRETEXT I/II, if the tumor is more than 1 cm away from the major branches of the hepatic vein or portal vein, surgery can be performed first (5); if the tumor has a wide range of invasion (PRETEXT III/IV), or if the tumor is invading or near to the major blood vessels, chemotherapy should be administered first because 90% of hepatoblastomas respond well to chemotherapy (6). It is also believed that the decision to remove the tumor should depend on whether a negative margin can be obtained (3). However, previous studies found that in clinical practice, many patients' tumors can also be completely removed by surgery despite close proximity to the main blood vessels after chemotherapy, and good prognoses can be obtained. And even when the resection margin is micro-positive, the overall survival rate is not worse (7). However, the above viewpoints were based on the analysis and follow-up of clinical data. At present, there is no report focusing on the border of the tumor from the perspective of pathology, and the microscopic condition of the tumor border is unknown.

To investigate the accurate invasion distance in the tumor border and to evaluate whether narrow resection margin (<1 cm) is acceptable, we designed this study. This study was approved by the Ethics Committee of the Affiliated Hospital of Qingdao University.

## Patients and Methods

### Patients

#### General Information

In total, 42 patients with hepatoblastoma who underwent surgery at the Affiliated Hospital of Qingdao University from January 2010 to December 2017 were selected. These 42 patients were divided into two groups according to the timing of operation: Group 1 patients underwent primary surgery immediately after diagnosis, and Group 2 patients received operation after two to four cycles of CHT on the basis of cisplatin and doxorubicin. There were 31.8% (7/22) patients in Group 1 and 95% (19/20) patients in Group 2 with a narrow resection margin. The general information of the patients is shown in Table 1. According to the Children's Oncology Group (COG) surgery guidelines (AHEP-0731), only patients with PRETEXT I or II can undergo surgery before chemotherapy. However, during the study period, the parents of six patients with PRETEXT III strongly requested the operation at diagnosis or they would abandon the treatment, and they signed a written consent. After preoperative evaluation, the tumor could be removed completely, and the residual liver volume was more than 40%, which could sustain their lives. Therefore, the operation was carried out first, and the prognosis was good after follow-up. These six cases also met the inclusion criteria of this study and were included in Group 1.

**Table 1:** General information of 42 patients with hepatoblastoma.

**Information**	**Primary surgery** **(*n* = 22)**	**Surgery followed CHT** **(*n* = 20)**
Gender		
Male	12 (54.5%)	13 (65%)
Female	10 (45.5%)	7 (35%)
Age		
~1 year	14 (63.7%)	4 (20%)
~2 years	4 (18.2%)	7 (35%)
~3 years	1 (4.5%)	4 (20%)
>3 years	3 (13.6%)	5 (20%)
PRETEXT system		
I	1 (4.5%)	0
II	15 (68.2%)	7 (35%)
III	6 (27.3%)	12 (60%)
IV	0	1 (5%)
Operation method		
Hemihepatectomy	10 (45.5%)	14 (70%)
Bisegmentectomy	1 (4.5%)	2 (10%)
Segmentectomy	6 (27.3%)	3 (15%)
Trisectionectomy	5 (22.7%)	1 (5%)
Pathological type		
Mixed epithelial–mesenchymal type	5 (22.7%)	7 (35%)
Epithelial type	17 (77.3%)	13 (65%)
Pure fetal type	7	11
Embryonal type	3	0
Hybrid	7	2
Resection margin		
>1 cm	15 (68.2%)	1 (5%)
0.5–1 cm	5 (22.7%)	4 (20%)
<0.5 cm	2 (9.1%)	15 (75%)

Our chemotherapy protocol followed *the Multidisciplinary Treatment Guideline for Chinese Children with Hepatoblastoma* developed *by* Chinese Children's Cancer Group (CCCG), and we used protocols from Société Internationale d'Oncologie Pédiatrique-Epithelial Liver Tumor Study Group (SIOPEL) and COG as references (2, 4, 6). The risk-stratification staging was made according to the standard of Children's Hepatic tumors International Collaboration (CHIC) (8). Patients in Group I received six to eight courses of chemotherapy after surgery with C5VD (cisplatin + 5-fluorouracil + vincristine + doxorubicin). In Group II, the patients in the intermediate-risk group underwent surgery after two to four courses of chemotherapy with a C5VD regimen, with a total course of six to eight; patients in the high-risk group underwent surgery after three to five courses of chemotherapy with cisplatin and doxorubicin regimens, with a total course of six to seven.

### Preoperative CT Three-Dimensional Reconstruction Assessment and Operation

Each patient underwent abdominal CT enhancement scans and was evaluated by the Hisense CAS 3D reconstruction system at the time of diagnosis (9); Group 2 patients underwent CT and three-dimensional (3D) reconstruction to assess tumor status also after two to four cycles of chemotherapy. Three-dimensional reconstruction was used to visually observe the size and location of tumors and to measure the distance between the tumor and the closest of the main blood vessels (the main trunk of the portal vein or hepatic artery or the main branch of the hepatic vein) before surgery.

**Figure 1:**
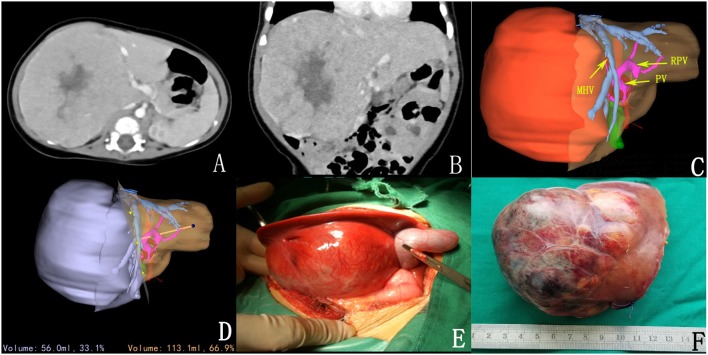
Preoperative assessment and surgical status. **(A,B)** CT sectional and coronal scan of tumor; **(C)** 3D reconstruction; **(D)** 3D reconstruction simulated the surgical section and calculated the residual liver volume; **(E)** intraoperative observation; **(F)** complete tumor resection.

**Figure 2:**
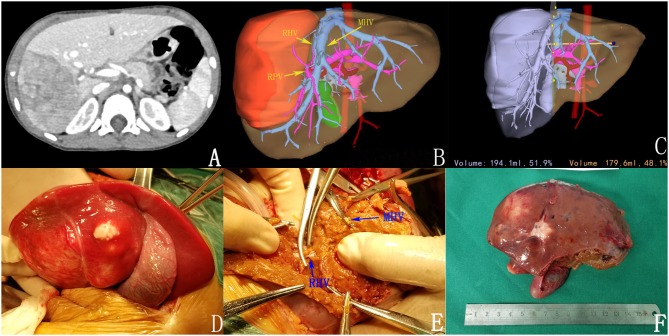
Preoperative assessment and surgical status. **(A)** Enhanced CT; **(B)** 3D reconstruction; **(C)** 3D reconstruction simulated the surgical section and calculated the residual liver volume; **(D,E)** intraoperative observation; **(F)** complete tumor resection.

**Figure 3:**
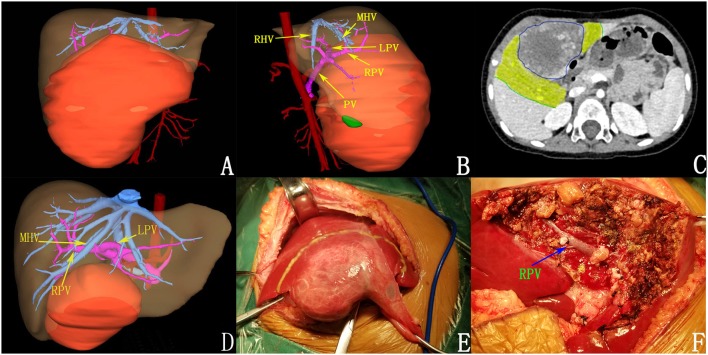
Preoperative assessment and surgical status of patients after chemotherapy. **(A,B)** 3D reconstruction before chemotherapy. **(C)** tumor volume decreased after chemotherapy (the yellow areas were tumor regression areas); **(D)** after chemotherapy; **(E)** intraoperative tumor morphology; **(F)** the tumor was completely resected.

The surgery was performed on the recommendation of the COG for the surgical treatment of hepatoblastoma and Chinese surgery guidelines for the hepatoblastoma (Figures 1–3) (2, 10). According to the general principle, the surgical margin should be 1 cm away from the tumor. In Group 1, five patients had tumor-to-vessel distance of 0.5–1 cm, but the primary surgery was still performed because the tumor could be completely removed after 3D simulation surgery; two patients with tumor-to-vessel distance <0.5 cm also received primary surgery as the tumor was small and could be completely removed. If the tumor after chemotherapy is still <1 cm away from the main blood vessel, the surgical margin should be as far away from the tumor as possible while ensuring vascular safety.

### Methods

First, follow-up was performed for each patient, mainly by telephone follow-up, outpatient review, or during hospitalization. After a monthly review for 0.5 years after surgery, the interval of the review was gradually extended, and telephone follow-up was conducted every 6 months. The survival status of each patient was recorded at each follow-up.

The resected specimens of patients with hepatoblastoma were visually and microscopically observed, and each section was completely scanned using a Nikon microscope section scanning system to present the overall morphology of the sections. Sections were assessed for whether a capsule was present around the hepatoblastoma, whether the tumor was invasive and exhibited breakthrough beyond the capsule, and whether there was evidence of micrometastasis in the paratumor liver tissue. Microscopic observation was performed by hematoxylin and eosin (H&E) staining, special staining, and immunohistochemical staining.

#### Pathological Sampling

**Figure 4:**
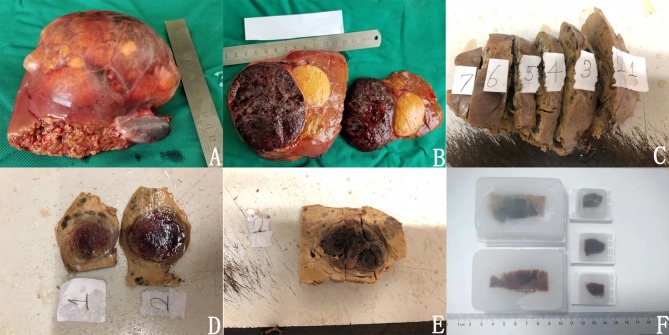
Methods of sampling and paraffin fixation. **(A)** *in vitro* specimens; **(B)** clear boundary between tumor and paratumor liver tissue; **(C)** after the specimen was fixed; **(D)** made large specimens; **(E)** complete sampling of tumor boundaries; **(F)** large specimen wax blocks and ordinary wax blocks had been embedded.

After the tumor was resected, it was cut along the maximum diameter perpendicular to the margin. The gross morphology, border morphology, and the distance between the tumor and the surgical margin were observed (Figure 4A). Then, the tumor was sectioned every 1 cm and fixed in 10% neutral buffered formalin for 12–24 h, and then samples were taken (Figures 4B,C).

We adhered to the following practice guidelines for the standardized pathological diagnosis of primary liver cancer in China (11): (1) In each section, samples were taken at the junction of the tumor and paratumor liver tissues at 12, 3, 6, and 9 o'clock locations. The proportion of tumor-to-paratumor liver tissue was approximately 1:2 in order to observe the tumor invasion and micrometastasis of paratumor liver tissue. (2) Liver tissue of <1 cm away from the surgical edge (proximal paratumor liver tissue or incisional edge) and more than 1 cm (distal paratumor liver tissue) were collected to observe whether the tumor had satellite nodules, dysplasia nodules, and liver tissue lesions (liver fibrosis and cirrhosis). (3) During sampling, the site number was recorded, and the size of the tissue block was (1.5~2.0) cm ^*^ 1.0 cm ^*^ 0.2 cm. In sections with small tumor diameters, the tumor and paratumor tissue were completely sampled as large specimens measuring 7.5 ^*^ 5.5 ^*^ 0.2 cm. The sampled specimens were fixed with 10% neutral buffered formalin solution for 12–24 h and embedded in conventional paraffin (Figures 4D–F).

#### Observation of the Capsule and the Capsule's Structure

Conventional pathological sections were made and then stained with H&E, and the tumor margins were observed under the microscope and scanned to observe whether the capsule was intact.

Three special staining methods were performed to determine the basic components of the capsule. Masson staining, Gomori silver staining, and Verhoeff staining were used to determine the presence of collagen fiber, reticular fiber, and elastic fiber; under the three special staining methods, the three fibers presented as blue, black, and black, respectively. The color of the capsules was observed by a high-power microscope to determine its basic composition.

#### Observation of Local Tumor Invasion and Breakthrough

Microscopically, H&E sections were continuously observed along the capsule to detect whether there were tumor invasion and capsule breakthrough.

#### Observation of the Micrometastatic Cancer Nests Outside the Capsule

Microscopically, H&E sections and scanned images were observed to investigate the metastatic cancer nests in paratumor liver tissue and to measure the distance between the cancer nests and tumors.

#### Immunohistochemical Staining Was Used to Determine the Border

To test whether there was abnormal protein expression in the paratumor liver tissue and to accurately determine the border of the tumor, we performed immunohistochemical staining of five proteins; the obtained liver tissue was collected as far away from the tumor as the normal control. Cytokeratin-8 (CK8), cytokeratin-19 (CK19), β-catenin, alpha-fetoprotein (AFP), and glypican-3 (GPC3) were selected for immunohistochemical staining. The expression levels of the five proteins in the tumor and paratumor tissues were observed by the microscope; the presence of abnormal protein expression in paratumor liver tissue was observed by using normal H&E staining.

#### Measurement of the Distance Between Cancer Nests and the Capsule

We first calculated the retraction rate of liver tissue resulting from the fixation and dehydration process. The length of tissue blocks was measured after tissue fixation and then again after dehydration, and the retraction rate of liver tissue during the entire process was calculated. If cancer nests were found in the paratumor liver tissue, the distance between the cancer nests and the capsule was measured on the scanned films. The actual distance from the cancer nests to the capsule was calculated according to the retraction rate.

## Results

### Patient Survival Status

All patients of Group 1 and 75% (17/20) patients of Group 2 acquired R0 resection. Long-term follow-up results were obtained in 18 of the 22 patients (81.2%) in Group 1 and 18 of 20 patients in Group 2 (90%). For the two groups, the average follow-up time was 59 (range: 15–104 months) and 46 months (range: 8–96 months), and the median follow-up time was 63 and 49 months.

In Group 1, one patient with a wide resection margin was found to have lung metastases 10 months after surgery and is at present still alive at 20 months after surgery. This patient was a 2-year-old boy who underwent right hemihepatectomy under PRETEXT II. The pathological type was epithelial (hybrid), and there were no cancer nests out of the capsule or intravascular microtumorous thrombi. In Group 2, one patient with a narrow resection margin died of multiple organ metastases 16 months after surgery. This case was a 3-year-old girl, PRETEXT III, and POST-TEXT III after four courses of chemotherapy. The surgical protocol was right trisectionectomy. The pathological type was epithelial type (hybrid), and there were no cancer nests out of the capsule or intravascular microtumorous thrombi. Other patients had no special events. The 3-year event-free survival rate and total survival rate of Group 1 were 94.4 and 100%, respectively; and those of Group 2 were 94.4 and 94.4%, respectively. The follow-up time and even-free survival rate are shown in Supplementary Figure 1.

### Observation of the Tumor Capsule

**Figure 5:**
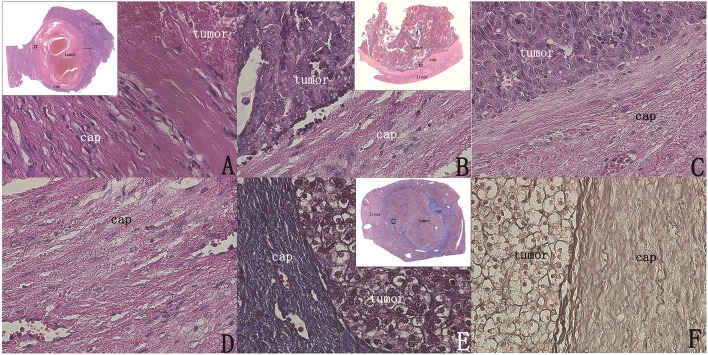
The intact capsule could be clearly shown by H&E staining and special staining (original magnification, 400×; inset, 40× scanned). **(A)** A 7.5 * 5.5 cm section showing the surrounding capsule of the tumor was intact. **(B)** Capsule integrity, no tumor breakthrough. **(C,D)** Clear image of the capsule. **(E)** Masson staining of the capsule showing obvious blue staining of the entire capsule, indicating that there were many collagen fiber components. **(F)** Verhoeff staining showing that the black fiber composition of the capsule layer is obvious, indicating that the capsule contains elastic fiber.

H&E sections of all tumors of the two groups were observed, and it was found that there was an intact capsule at the edge of all hepatoblastomas, which divided the tumor and the paratumor liver tissue into two parts with clear boundaries (Figures 5A,B). The capsule was observed as a lamellar structure surrounding the tumor tissue, containing fibrous cells with few nuclei. H&E staining showed mainly eosinophilic red staining, and a small number of lymphocytes were observed between the fibrous cells in each layer. The structure of hepatic lobules near the capsule differed from the structure of normal liver tissue. Abnormal proliferation of bile ducts was observed and showed an irregular structure. The normal tubular structure was not present, but no obvious tumor cells were observed (Figures 5C,D).

Under Masson staining, collagen fibers appear blue. Following Masson staining of the sections, it was found that the capsule presented with a clear bright blue color, indicating a large number of collagen fibers (Figure 5E). By observing elastic fiber and reticular fiber staining of the sections, it was found that these two fiber components were also present in the capsule, but their levels were significantly lower than those of collagen fibers (Figure 5F).

### Observation Results of Capsule Invasion and Breakthrough

**Figure 6:**
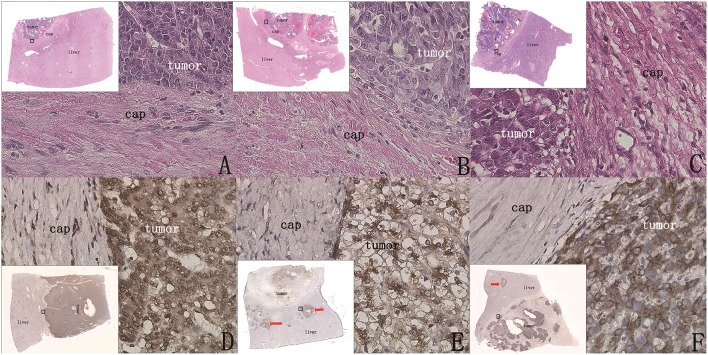
Whether there were cancer nests outside the capsule could be clearly shown by H&E staining and immunohistochemical staining (original magnification, 400×; inset, 40× scanned). **(A–C)** (H&E) The capsule was intact, the boundary between tumor tissue and paratumor liver tissue was clear, and there was no tumor infiltration in the paratumor liver tissue. **(D)** (GPC3 staining) Tumor coloration is clearly shown and is significantly different in paratumor liver tissue, and there are no cancer nests outside the capsule. **(E,F)** (GPC3 staining) Cases after chemotherapy; cancer nests were found outside the capsule (red arrow).

Through the examination of the sections stained with H&E and immunohistochemical staining, we found that the capsules of all the 42 patients were all intact, with no tumors invading directly into the liver tissue through the capsule (Figures 5A,B, 6A–C). Although no patients exhibited tumor invasion through the capsule, three patients (13.6%) in Group 1 and two patients (10%) in Group 2 were found to have intravascular microtumorous thrombi outside the capsule.

### Observation Results of Extracapsular Micrometastatic Cancer Nests

In Group 1, 90.9% (20/22) of patients had no micrometastatic cancer nests outside the capsule (Figures 6A–D). In Group 2, 75% (15/20) had cancer nests outside the capsule (Figures 6E,F). It was observed that all the cancer nests of these patients were located in the chemotherapy regression area (the tissue area that was the tumor before chemotherapy and replaced by liver tissue after chemotherapy as the tumor volume decreased), and it was speculated that the extracapsular cancer nests were the residual cancer nests after chemotherapy.

### Observation Results of Immunohistochemical Staining

The normal liver tissues were observed, and the expression levels of CK8, CK19, β-catenin, and AFP were all low, whereas GPC3 was not expressed. CK8 was mainly expressed in hepatocytes in the portal area and in the cell membrane and cytoplasm. CK19 was mainly expressed in bile duct epithelial cells. β-Catenin was mainly expressed in the cell membrane and weakly expressed in the cytoplasm. AFP was also weakly expressed in the membrane and cytoplasm of liver tissue.

The positive expression rates of GPC3, CK8, CK19, β-catenin, and AFP in the tumor tissue of these 42 patients were 100% (42/42), 88.1% (37/42), 92.9% (39/42), 92.9% (39/42), and 64.3% (27/42), respectively. In the tumor tissue with positive expression, protein expression was significantly increased, and ectopic expression was observed with high expression in the cell membrane and cytoplasm.

In the 25 patients without cancer nests outside the capsule (20 patients of Group 1 and five patients of Group 2), there was no abnormal expression of these five proteins in the paratumor liver tissue; CK8, CK19, AFP, and β-catenin were weakly expressed and in the expected locations, and GPC3 was not expressed in the paratumor liver tissue, which clearly indicated the tumor boundary (Figures 6D–F). These results indicated that there was no tumor micrometastasis outside the capsule, and there was no abnormal expression of these five proteins in the paratumor liver tissue.

In the 17 patients with cancer nests outside the capsule (two of Group 1 and 15 of Group 2), small cancer nests were scattered in the paratumor liver tissue, and protein expression of these small cancer nests was consistent with that of the tumor tissue; however, the protein expression of liver tissue outside the cancer nests was not abnormal.

### The Distance Between Paratumor Cancer Nests and Tumor Capsule

The regression rates of pathological specimens throughout the sample preparation process were calculated to be 6.8%. The Nikon microscopy scanning and measurement system were used to accurately measure the distance between the farthest end of cancer nests and the capsule in the 17 patients with extracapsular cancer nests. After the conversion of the retraction rate, the actual maximum distance between the cancer nests and capsule was 4.6 mm of Group 1 and 9.6 mm of Group 2.

## Discussion

Many controversies about the treatment of hepatoblastoma still remain. Neoadjuvant chemotherapy combined with surgical treatment is the standard treatment method for hepatoblastoma, and surgical treatment is the basis of comprehensive treatment (12). However, studies have found that the volume of hepatoblastoma will decrease after neoadjuvant chemotherapy, but the distance between the tumor and the main blood vessels has not been significantly reduced (13). If it is insisted that the tumor should be removed at a distance greater than 1 cm away from the large blood vessel, then many patients will be ineligible for operation. The most direct basis for the safe range of the resection margin should be the microscopic observation of the tumor border. From this perspective, we attempted to study the microscopic boundaries of hepatoblastoma and to provide pathological evidence for determining the optimal surgical resection range.

The correct sampling method is the basis for obtaining reliable results. In this study, large specimens and small specimens were combined to obtain complete tumor margins as distant as possible. The advantage of large specimens is that there are enough regions to show the situation outside the capsule, and paratumor liver tissue is also sampled at different distances from the capsule to study whether there are residual cancer nests at different distances to ensure the accuracy of the research results. Only when the tumor boundary is completely sampled can its condition be fully observed to avoid sampling errors.

H&E staining of tissue sections can show the morphology of the tumor tissue and capsule, but it has the potential to miss the small cancer nests outside the capsule. Immunohistochemical staining of five proteins clearly shows the difference in expression levels of proteins in tumor tissue and paratumor liver tissue, and it can accurately show the presence of cancer nests outside the capsule (14). In terms of tumor boundary display, the GPC3 protein is only expressed in tumor tissue but not in liver tissue, so it has the best display effect and can clearly indicate the presence of cancer nests in the paratumor liver tissue.

In our study, we found that there was an obvious fibrous capsule around the hepatoblastoma, which blocked the local invasion of the tumor, and there were no infiltrating nests outside the capsule. This is markedly different from the border invasion of hepatocellular carcinoma. In some pathological types of hepatocellular carcinoma, the tumor also has a capsule (15). However, hepatocellular carcinoma cells can still break through the capsule and grow in an infiltrating manner, invading the periphery along the blood vessels similar to “tree roots” (16, 17); as such, infiltration is not related to the presence of a capsule (18). Such extracapsular infiltration can reduce the survival rate of patients with narrow surgical margins (<1 cm) (19), so when resection of hepatocellular carcinoma is performed, the surgical margins should be more than 1 cm away from the tumor. However, hepatoblastoma is different. According to our study, there is no invasion of cancer nests outside the tumor capsule of patients with hepatoblastoma without chemotherapy, and children with narrow surgical margins (<1 cm) can also obtain a good prognosis, which is consistent with the follow-up results in other studies (20, 21). Therefore, radical surgery can be performed more actively in hepatoblastoma surgery even if the tumor is <1 cm away from the main blood vessels.

In patients following chemotherapy, tumor volume was significantly reduced, and the tumor tissue in the tumor regression area was replaced by liver tissue. Although the boundaries of the tumor are clearly defined upon imaging, our study found that the cancer nests still remained in the tumor regression area. This provides a pathological basis to determine the distance of the surgical margin. When patients with hepatoblastoma undergo surgery after chemotherapy, the margin may be positive if the surgical margin is not large enough, and postoperative chemotherapy and close follow-up are required; however, some studies show that a good prognosis can be obtained even if the surgical margin is positive (22). In our study, the farthest distance between the cancer nests and the capsule was 1 cm, but owing to the small number of cases, the specific tumor regression area requires further study.

Hepatoblastoma, although large in size, has a capsule and a high resection rate. Preoperative chemotherapy can reduce tumor volume and blood supply to reduce complications and improve resection rates. Therefore, for some large tumors that cannot be resected in one stage, efforts should be made to perform delayed surgical resection after active chemotherapy. Computer-assisted surgery (CAS) system can greatly aid in the resection of liver tumors (23). CAS can conduct 3D reconstruction of two-dimensional CT/MRI image data before surgery, clearly display the shape and anatomical relationship of the vascular system in the liver, and restore the 3D anatomical conformation of the lesion and the surrounding vascular structure; this allows for accurate localization and evaluation of the lesion, and it helps to formulate a reasonable surgical plan. CAS can improve the radical operation, safety, and resection of lesions, and it reduces the incidence of liver failure and other complications.

Narrow resection margin is acceptable in hepatoblastoma surgery, which does not make the prognosis worse. The capsule of hepatoblastoma can limit the local infiltration of the tumor, so hepatoblastoma can be more actively treated with radical surgery. However, following chemotherapy, patients may still have cancer nests in the tumor regression area.

## Data Availability Statement

The datasets generated for this study are available on request to the corresponding author.

## Ethics Statement

The studies involving human participants were reviewed and approved by Ethics Committee of the Affiliated Hospital of Qingdao University. Written informed consent to participate in this study was provided by the participants' legal guardian/next of kin.

## Author Contributions

GS, QD, LW, and JZ: study conception and design. BW, XZ, and FW: data acquisition. GS and JL: analysis and data interpretation. GS and QD: drafting of the manuscript. QD: critical revision.

### Conflict of Interest

The authors declare that the research was conducted in the absence of any commercial or financial relationships that could be construed as a potential conflict of interest.
