# Supplementary material for: Clinical and Pathological Study of Tumor Border Invasion—Is Narrow Resection Margin Acceptable in Hepatoblastoma Surgery?
Source: Front Med (Lausanne). 2020 Mar 4;7:59. doi: 10.3389/fmed.2020.00059 (PMC7064447; doi:10.3389/fmed.2020.00059)
Supplement: Supplementary file 1 [file Data_Sheet_1.pdf]

Supplementary Material

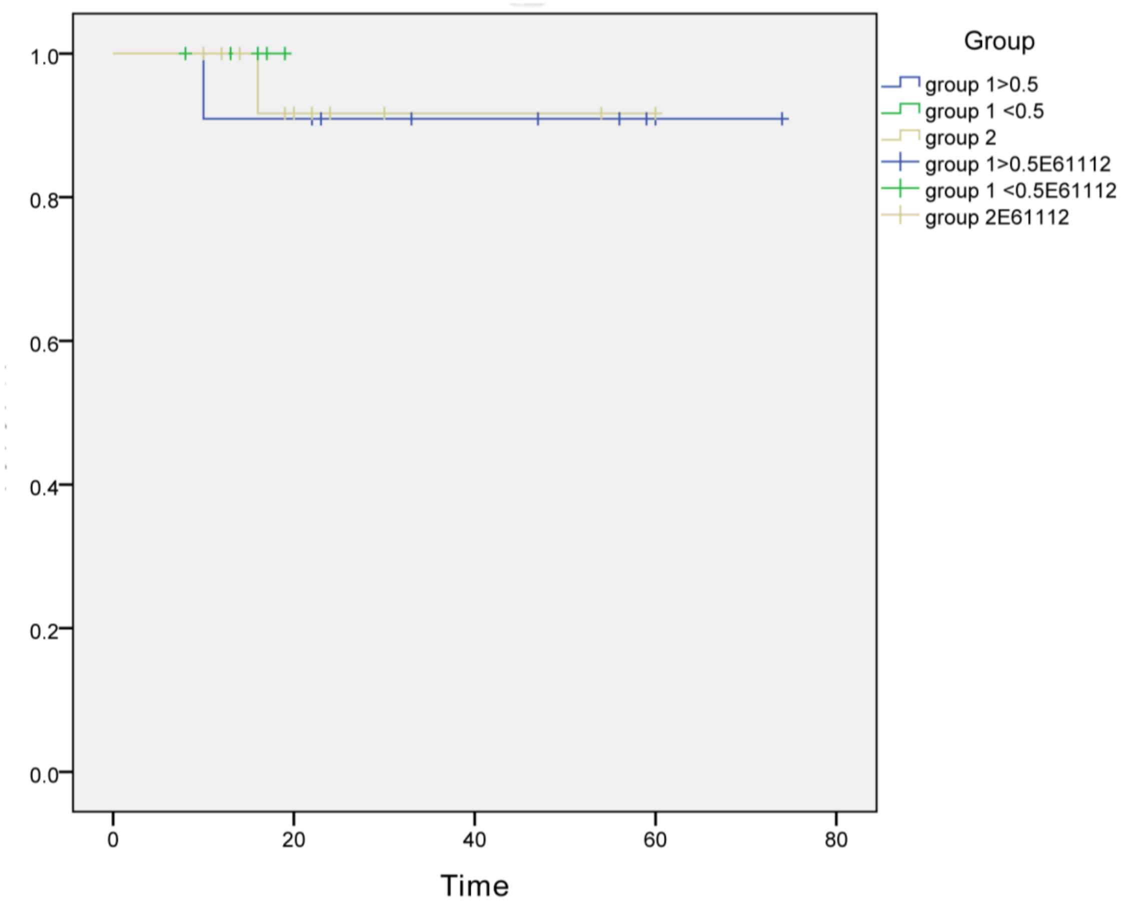

Supplementary Fig 1. The event-free survival rate curve of the wide resectable margin patients of Group 1, narrow resection margin patients of Group 1 and patients in Group 2. The event-free survival rate is not statistically different in the three parts of patients.
